# Changes in Physical Activity, Physical Fitness and Well-Being Following a School-Based Health Promotion Program in a Norwegian Region with a Poor Public Health Profile: A Non-Randomized Controlled Study in Early Adolescents

**DOI:** 10.3390/ijerph17030896

**Published:** 2020-01-31

**Authors:** Sabrina K. Schmidt, Michael S. Reinboth, Geir K. Resaland, Solfrid Bratland-Sanda

**Affiliations:** 1 Faculty of Humanities, Sports and Educational Science, University of South-Eastern Norway, Pb 235, 3603 Kongsberg, Norway; michael.reinboth@usn.no (M.S.R.); solfrid.bratland-sanda@usn.no (S.B.-S.); 2 Center for Physically Active Learning, Faculty of Education, Arts and Sports, Western Norway University of Applied Science, Campus Sogndal, 6856 Sogndal, Norway; Geir.Kare.Resaland@hvl.no

**Keywords:** adolescents, school-based physical activity, physically active academic lessons, intervention, well-being

## Abstract

The purpose of this study was to examine the changes in physical activity (PA), physical fitness and psychosocial well-being in early adolescents following implementation of a school-based health promotion program in secondary schools. Methods: Six municipalities in Telemark County, Norway, were recruited into intervention (6 schools) or control groups (9 schools). A total of 644 pupils participated in the study (response rate: 79%). The schools in the intervention group implemented the Active and Healthy Kids program, where the PA component consisted of (1) 120 min/week of physically active learning (PAL) and (2) 25 min/week of physical active breaks. Furthermore, both the intervention and control schools carried out 135 min/week of physical education. The primary outcome was PA. Secondary outcomes were sedentary time, physical fitness, subjective vitality and health-related quality of life (HRQoL) in five domains: physical health, psychological well-being, parent, peers and school. Results: There was a group x time effect on school-based PA (*p* < 0.05), but not total PA, as well as on physical fitness (*p* < 0.05) and vitality (p < 0.01). In girls, there also was a group x time effect on three out of the five domains on HRQoL (*p* < 0.05). Conclusions: A multi-component, school-based health-promotion program with emphasis on the use of PAL led to positive changes in school-based PA levels. Furthermore, positive changes were seen in physical fitness, vitality and HRQoL among early adolescents in a county with a poor public health profile. This might have implications for the development and promotion in schools of general health and well-being throughout adolescence.

## 1. Introduction

There is a well-documented overall positive effect of physical activity (PA) on mental health and well-being [1]. Indeed, PA is widely recognized as an important determinant of physical and psychosocial health and development among children and adolescents [1,2,3]. Although most adolescents report good mental health and quality of life, the prevalence of mental health challenges in this age group is increasing [4]. Norwegian national representative data showed that from 2011 to 2016 the levels of mental health symptoms increased by 24% in adolescent girls [5], and the prevalence of diagnosed mental illnesses in adolescent girls increased by 40% [5]. Similar trends have been reported in other countries [6,7]. Simultaneously, there is a steady decrease in PA levels from childhood to adolescence in Norway [8]. Many adolescents in Norway and other Western countries are insufficiently physically active to benefit from the positive factors of PA, as only about 50% of 15-year-olds meet the recommendation for daily PA [9,10,11]. The World Health Organization (WHO) have launched a PA action plan [12] aimed at reducing physical inactivity and a comprehensive school-based PA program [13]. In addition, the WHO and UNESCO launched a global standard for health promotion in schools because schools are identified as a key arena for promoting health, well-being and a healthy lifestyle [14].

Although Norway as a country has relatively high ratings on public health indicators compared with other countries [15], Telemark County in Norway has a poor public health profile with a higher prevalence of mental health challenges and shorter life expectancy than average in Norway [16]. To this end, the Telemark County Council initiated the Active and Healthy Kids program in 2016. This is a school-based, health-promoting program, which aims to improve living conditions for children and adolescents through increased school-based PA, healthier school meals and an improved psychosocial environment. One of the components, school-based PA, is built on the WHO’s comprehensive school-based PA program. PA in classrooms/physically active learning (PAL) is one important component and strategy to reach higher PA levels among children and adolescents [17]. PAL is the use of PA as a pedagogical tool for learning academic content in other subjects than physical education (PE) [18]. This strategy has been used by several school-based PA interventions [19,20,21]. Most studies have examined the use of PAL in children [22]; less is known about how early adolescents in secondary schools will respond to such an intervention. Studies on school-based PA and adolescents often use other strategies for increasing school-based PA, such as increasing the number of PE lessons and/or active breaks/recess [23,24]. 

The way we approach mental health has changed, and the concept of salutogenesis represents a shift from preventing mental health challenges, such as anxiety and depression, to promotion of well-being and quality of life [25]. Using this perspective, positive emotions in early adolescents are linked to fewer relational problems and better work functioning in adulthood [3]. The development of life skills, such as good health, has also been acknowledged as important and is included as part of the OECD Education 2030 [26]. The effects of school-based PA interventions have mostly focused on improving physical fitness, reducing the risk of non-communicable diseases, increasing learning and decreasing mental-health challenges [20,27,28,29,30,31]. Less knowledge and attention have been given to the potential of school-based PA to improve health-related quality of life, vitality and well-being associated with increased school-based PA [32]. Subjective vitality emerges as one component under the umbrella of well-being [33], and is conceptualized as a psychological sense of aliveness, enthusiasm and/or energy. Nix and colleagues highlight that vitality has a regenerative capacity that is not necessarily representative of happiness but of broad emotional states, which is a common conception of well-being [34]. Baily and Colleagues [35] underline that positive development associated with PA does not occur automatically; PA’s contribution to well-being is conditional to the context and especially the social climate generated by, e.g., educators [35]. To evaluate and get a more comprehensive picture of how a school-based PA program with PA and academic content combined affects adolescents’ health and well-being, we need to not only investigate the impacts on more objective outcomes like PA, cardiovascular indicators and aerobic fitness, but also well-being and sedentary time.

As mentioned, schools have been identified as a key setting to ensure adequate PA levels; however, a recent review from Love et al. [36] finds that current school-based efforts do not positively impact young people’s PA across the full day. When looking at PA in school time, a recent meta-analysis from Norris et al. [22] looked specifically at interventions using PAL and concluded that there is a positive effect of PAL on PA compared to a normal subject lesson. When looking at overall PA they found a non-significant or small effect [22]. Because of a lack of results on PA across the full day, Love et al. [36] recommend that, for now, school-based PA interventions should continue to be conducted in a research context. Further, Norris et al. states that more studies should include secondary schools and assessment of a more diverse range of health outcomes [22]. This study aims to examine whether the Active and Healthy Kids program led to changes in PA, sedentary time, physical fitness, well-being and health-related quality of life (HRQoL) in early adolescents. The research questions were as follows: (1) do PA, physical fitness, well-being and HRQoL change in early adolescents following a school-based, health-promoting program? and (2) are there gender differences in the changes observed following the Active and Healthy Kids program?

## 2. Materials and Methods

### 2.1. Participants and Study Design

**Figure 1:**
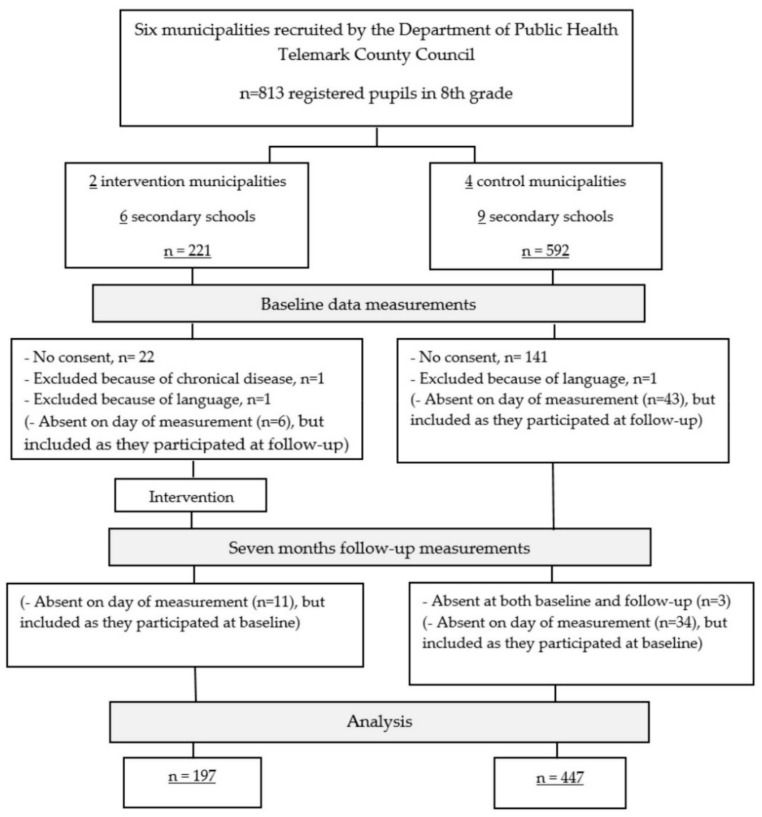
Flow of pupils and study design.

To evaluate the implementation of PA in schools by the Telemark County Council, we conducted a quasi-experimental seven-month study using a pre–post control group design. In Norway, the secondary school consists of three years referred to as 8th, 9th and 10th grade, and pupils are between 13 and 15 years of age. Inclusion criteria for this evaluation were enrolment as a pupil in the 8th grade in the 2017/2018 school year, and being in a public secondary school in the six municipalities that were enrolled for implementation of the Active and Healthy Kids program by Telemark County Council. One rural and one urban municipality implemented the program in 2017/2018 and therefore served as the intervention group, whereas three rural municipalities and one urban municipality planned to implement the program in 2018/2019 and hence served as the control group. The 1:2 ratio for intervention and control municipalities is therefore a pragmatic approach due to the naturalistic setting of the implementation. The six municipalities had a total of fifteen secondary schools and 813 pupils registered in 8th grade, all of whom were invited to participate (Figure 1).

The research group provided oral and written information about the study to school principals and staff, and the primary teachers for each included class distributed written information about the study to the pupils and their parents on behalf of the research group prior to data collection. Written consent was obtained from parents for all included pupils. Data collection was performed during one school day, and pupils absent from school that day were unable to participate; however, they were included if they provided data at one of the measurement times. A total of 644 pupils were included. The major reasons for non-participation were lack of consent from a pupil or a parent and absence on the day of measurement. Exclusion criteria for participation were language barriers and/or injuries and illness that influenced the assessment of physical fitness and PA. There were specific challenges with recruitment and retention regarding the accelerometer to measure PA. At baseline, 484 pupils attained the minimum four days of valid accelerometer recordings for a full day, while 66 recordings were excluded because of invalid wear time. At follow-up, 329 pupils had sufficient wear time and 193 were excluded because of invalid wear time. During school hours (09:00–14:00) more students were included because of valid wear time in that period: 539 pupils at baseline and 473 pupils at follow-up. Lost accelerometers (10 at baseline and 6 at posttest) accounted for a small proportion of missing data with the majority due to refusal to wear the monitor and to low wear time. This study was conducted in accordance with the Helsinki Declaration, approved by the Norwegian Data Protection Services (ID number 54327) and registered in ClinicalTrials.gov (NCT03906851). The data was collected at two times at the local school; baseline September 2017 and follow-up April/May 2018.

### 2.2. Intervention Program

The Active and Healthy Kids program is a health promoting multi-component program, developed and implemented by the Telemark County Council to increase learning, well-being and health for pupils in elementary and secondary schools. It is conducted within a socio-ecological framework that recognizes that PA behavior is influenced by multiple levels [37]. Further, it is based on a salutogenic perspective [25] and basic principles of the self-determination theory [38], wherein the facilitation of the three basic psychological needs (Autonomy, Relatedness and Competence) promote intrinsic motivation and well-being. However, no specific motivational training of teachers was performed. The program was pilot tested at one secondary school in 2015–2016 before the experimental study began. The program consists of three main strategies to reach the overall goal: (1) healthy diet, (2) awareness of important lifestyle factors, and (3) increased PA. In this paper, we will focus on the PA strategy, and therefore the other strategies will only be briefly described.

The healthy diet strategy introduces national guidelines for food and meals in schools, providing varied school cafeteria menus and reducing unhealthy and sugar-rich foods and drinks in the school cafeteria. A trained cook facilitated the training of cafeteria staff. The pupils were informed about the importance of a healthy lunch packet and the school focused on creating a social eating environment in the lunch break. Awareness of important lifestyle factors is based on knowledge and experience with the associations between PA, a healthy diet, learning, health and well-being.

The strategy to increase PA is based on a modified Active Smarter Kids model, where PA activity is being used as a teaching tool for repetition and overlearning of already-known school material [20,39]. This model offered a solution to meet the goal of 60 min of daily PA through teacher-led activities. It consisted of three components: PAL (135 min/week), physically active 5-min breaks (25 min/week) and PE as usual (135 min/week). Adding the weekly minutes of physical activities divided across the school week of five days meant that the pupils would be physically active 59 min/day. PAL is a normal subject lesson planned and led by the classroom teacher where the whole lesson, or part of the lesson, is performed outside the classroom, often in the schoolyard, where pupils are physically active while working with school material, mainly repetition of already-learned material. Furthermore, PAL is an integrated part of the teaching, not a break; it consists of teacher-implemented academic lessons that utilize moderate to vigorous movement in the review or teaching of core academic content. PAL is mainly used in conjunction with the three major subjects taught in secondary schools: English, Math, and Norwegian, without reducing educational time. The teachers were trained in PAL as described in Table 1. Furthermore, each school appointed a resource teacher, who took part in resource teacher gatherings once every semester.

The program is a collaboration between teachers, school leaders, project leaders at the municipality, the school health service and the school nurses. The Health Department at Telemark County Council is the program leader and has facilitated competence development and training for all partners. 

### 2.3. Instruments and Measures

#### 2.3.1. PA and Sedentary Time

PA was objectively assessed using accelerometers (ActiGraph GT3X+, LLC, Pensacola, FL, USA) at 10 s epoch intervals. Each participant was fitted with an accelerometer in an elastic belt around their waist placed on the right hip, worn for four consecutive days (two weekdays and two weekend days). Participant were instructed to wear their accelerometers during the whole day except during water-based activities or while sleeping. Accelerometers were initialized to start recording at 6 a.m. on the day after they were distributed. Criterion for a valid day was set as a wear time of ≥ 480 min/day accumulated between 06:00 and 24:00 and ≥ 2 (out of 4) days were applied as criteria for a valid measurement for a full day. All sequences of ≥ 20 min or more of consecutive zero counts from each subject recording were excluded and defined as non-wear time, as this implies time where participants did not wear the accelerometer [40]. These criteria were the same as used in the PA among Norwegian Children study [11]. For a valid school day the criteria was set to a wear time of ≥ 180 min/day accumulated between 09:00 and 14:00, same as in the Active Smarter Kids study [39], with a total of ≥ 1 (out of 2 days). We used the ActiLife software (ActiGraph, LLC, Pensacola, FL, USA) to initialize the monitors and to download the accelerometer files. The outcomes for total PA were counts per minute (cpm) from the accelerometers’ vertical axis (cpm axis 1). Sedentary time was defined as all activities < 100 cpm, a threshold that corresponds with sitting, reclining or lying down [41,42]. Evenson [41] was used for defining cutoffs for sedentary time in min/day (0–100 cpm) and for moderate-to-vigorous PA (MVPA) (≥ 2296 cpm). The Evenson cut-off points have shown acceptable classification accuracy for activity intensities among children [43]. We analyzed all accelerometer data by using ActiLife software (ActiGraph, Pensacola, FL, USA). Additionally, we reported the proportion of participants who achieved the guideline PA level on a daily basis (a minimum of 60 min/day of MVPA).

#### 2.3.2. Cardiorespiratory Fitness

The Andersen test, which is found to provide reliable and valid data on a group level [44], was used for assessing cardiorespiratory fitness. The Andersen test is a 10 min intermittent running field test: Pupils ran from one line to another (20 m apart) in periods of 15 s work and 15 s rest [45]. The test was indoors on a wooden or rubber floor. Distance covered in meters was recorded as the outcome for the analysis.

#### 2.3.3. Strength

The standing long jump test (SLJ) was used to measure lower- and upper-body muscular strength/power. The SLJ is a practical, time-efficient and cheap method of assessing the muscular fitness of children and adolescents in a school environment [46]. The pupils had to stand with both feet behind a line and were allowed to swing their arms and bend their knees to create momentum. The pupils were instructed to jump as far as possible landing on both feet, without falling. Two attempts were allowed for each pupil and the best attempt in terms of longest distance recorded was the outcome. Distance was measured from take-off line to the back of the heels.

#### 2.3.4. Anthropometric Measurements

Height and body weight were measured wearing light clothing and without shoes. Height measurements were collected using a wall-mounted standardized or stadiometer placed at the school nurse’s office, and was measured to the nearest 0.1 cm. Body weight was measured to the nearest 0.1 kg using an ADE electronic weight (ADE, Hamburg, Germany), and electronic scale weights belonging to the school nurse’s office at each school. BMI was calculated as weight in kilograms divided by height in squared meters (kg∙m^−2^).

#### 2.3.5. HRQoL

To obtain information regarding pupils’ perception of general well-being we used the KIDSCREEN-27 questionnaire. This was developed to construct a shorter version of the original KIDSCREEN-52, and consists of the five domains “Physical well-being” (5 items), “psychological well-being” (7 items), “autonomy and parent relationship” (7 items), “peers and social support” (5 items) and “school environment” (5 items). KIDSCREEN is a multi-dimensional, widely used and validated instrument that covers physical, psychological, social and behavioral components of well-being in children and adolescents aged 8-18 years [47,48]. Higher scores indicate better HRQoL.

#### 2.3.6. Well-Being and Vitality

Subjective Vitality Scale: The concept of subjective vitality is developed within the framework of the self-determination theory (SDT) [49]. It refers to a person’s energy and its relation to psychological well-being and has been defined as one’s conscious experience of possessing energy and aliveness [50]. Subjective vitality has shown associations with self-actualization, self-determination, mental health and self-esteem, and the subjective feeling of aliveness and vitality potentially represents a significant indicator of personal well-being. We have used The Subjective Vitality Scale by Ryan and Frederick [50], a short instrument used to measure vitality consisting of 7 items (e.g., I feel full of energy). Responses were given on a 7-point Likert scale from 1 (strongly disagree) to 7 (strongly agree). We have used the Individual Difference Level Version that ask participants to respond to each of the items by indicating the degree to which the item is true for them in general in their life. In previous research, this scale has been found to be valid and reliable [50,51].

#### 2.3.7. Demographic Characteristics

Parental education level has been stated as the most fundamental indicator for socio economic status (SES) [52,53]. The parents of each participant were thus asked to classify their completed educational level within one of the five following categories: “lower secondary school”, “vocational school”, “high school”, “higher education, undergraduate level” or “higher education, graduate level”. The highest educational level from each family was used for the analyses. Parental education level at the intervention group showed lower secondary school 2.4%, vocational school 14.4%, high school 10.4%, undergraduate level 51.2% and graduate level 21.6% (91 families provided data). Parent education at the control group showed lower secondary school 1.1%, vocational school 16.5%, high school 13.3%, undergraduate level 45.5% and graduate level 23.8% (273 families provided data).

### 2.4. Statistical Analysis

SPSS 26.0 was used for the statistical analyses. Little’s MCAR test showed that data were not missing completely at random (*p* < 0.001). Analysis of the missing values pattern showed that the dataset was non-monotone, hence we used multiple imputation of data to complete the dataset for the baseline analysis and for the t-test analysis from baseline to follow-up. Independent sample t-test and chi-square were used to analyze differences between the intervention and control groups at baseline. All dependent variables were standardized prior to analysis. A linear mixed model was conducted for all dependent variables to consider cluster random effects. The effects of the intervention were assessed by examining 2-way interactions (group x time) with a nested random effect of each subject of school with Bonferroni corrections. The minimum significance level was set at *p* < 0.05. 

## 3. Results

A total of 644 pupils provided written consent to participate: 197 pupils enrolled at the intervention schools and 447 pupils from control schools, with an even proportion of male and female participants across intervention and control schools (*p* > 0.05). Descriptive baseline characteristics of the intervention and control groups are presented in Table 2. The intervention and control group did not statistically differ with respect to demographic data (gender, age and SES), but the intervention group reported a significantly greater baseline level of the HRQoL domain “school environment” (*p* = 0.018).

### Changes in Intervention and Control Group at Baseline and Follow-up

Changes between the groups were observed for school-based PA level, physical fitness, HRQoL and vitality (Table 3). The control group increased sedentary time for full day, and there was a tendency of such an increase also in the intervention group. The Actigraph monitor measurements of time daily spent in MVPA showed that 36% of the adolescents adhered to the PA recommendations of a minimum of 60 min/day of MVPA. This adherence rate did not change between baseline and follow-up.

The control group showed a reduced score on HRQoL psychological well-being, peers and social support and school environment, as well as a reduced score on vitality (Table 3). Both the intervention group and control group had an improved score on HRQoL autonomy and parent support with greater improvements in the intervention group (Table 3).

The linear mixed model showed no effects on total PA level, but there was a group x time effect for the total sample on school-based PA level, physical fitness and vitality (Table 4). When analyzing boys and girls separately, there was a group x time effect on school-based PA for the boys (Table 5), and a group x time effect on physical fitness, HRQoL physical well-being, psychological well-being and autonomy and parent relationship, as well as on vitality for the girls (Table 6).

## 4. Discussion

The main findings were the positive effects on school-based PA levels and the lack of effects on total PA level. Furthermore, we found positive effects on physical fitness and vitality in the total sample, and on vitality and domains of HRQoL among the girls. We found negative effects on sedentary time among the girls.

The program showed positive effects on school-based PA level across a full day. This is in accordance with findings from Dobbins et al. [54], who reported that school-based PA interventions lead to more engagement in MVPA during school hours. In our study, the intervention group is stable over time in minutes spent in MVPA during school hours, where the control group declines. Similar results were found by Gammon et al. [55] who implemented PAL in secondary schools [55]. Because of the general decline in PA levels from child to adolescent, it is argued that interventions that attenuate PA decline could be considered effective [56]. However, the analysis showed that only boys had significantly increased time spent in MVPA. This supports results from other studies, which found that both children and adolescent boys were more involved in MVPA than girls [57,58]. Increased PA during school supports finding by Norris et al. [22]. The lack of effects on total PA level supports the results of Love et al. [36], who found no positive effect of school-based PA across the full day. It must be emphasized that previous studies have mostly examined populations of children in elementary school, and these results are not necessarily transferable to adolescents in secondary schools. Potential challenges for PAL in secondary schools are age, pubertal status, a more advanced curriculum and learning outcomes, as well as a focus on academic testing. In addition, the general PA level is lower among adolescents compared to children [8] and this might require more advanced skills in motivating and encouraging adolescents to actively participate in PAL. Furthermore, adolescence is a time period in life with large dropout rates from organized sports [59], and this requires even greater efforts in order to improve total PA levels. Therefore, it can be argued that the observed effects on school-based PA level were more difficult to achieve than in the previous findings from elementary school. It must also be noted that the objective measurement of PA was conducted for four days (two weekdays plus Saturday and Sunday), and not for an entire week. This might have influenced the total PA level, since the inclusion of more weekdays in the measurement period would have provided measurement of more school hours as well.

As shown in another Norwegian school-based PA intervention that included PAL [57], adolescents at the intervention schools increased cardiorespiratory fitness compared to the control schools. The intervention group also showed increased strength. This indicates that the activities in the school-based PA with emphasis on PAL had sufficient intensity and movement activities to achieve improvements in cardiorespiratory fitness and strength, resulting in overall improvements in physical fitness. Cardiorespiratory fitness is a powerful marker for health as it is associated with, among others, total abdominal adiposity, cardiovascular disease risk factors, positive effects on depression, anxiety, mood status and self–esteem [60]. Hence, Ortega and colleagues conclude that health promotion policies and PA programs should be designed to improve cardiorespiratory fitness [60]. However, the findings contradict those reported in the review of Norris et al. [22], who conclude that PAL was not sufficient to improve cardiorespiratory fitness [19,61,62]. 

The effects on vitality were shown by an actual reduction in vitality in the controls and a stability in vitality in the intervention group. The time period of early adolescence has previously shown that well-being can be impaired during this time [63], hence the potential of implementing a school-based PA intervention with emphasis on PAL to prevent such impairments are very interesting. 

There is compelling evidence that regular PA can have a positive effect on emotional well-being, especially the well-being of children and adolescents. PA is also linked to a variety of mental health outcomes [1], yet this is to our knowledge the first study to show this effect using a school-based health promotion program with emphasis on PAL on adolescents. The positive findings on vitality and well-being indicate that the program holds some qualities that can improve the fulfilment of the three basic psychological needs that lead to intrinsic motivation and well-being, where vitality is an indicator [49]. The specific effects on vitality and HRQoL observed among girls are interesting as this is the gender group where highest rates of mental health challenges are reported. The findings can be seen in relation to, e.g., Harrington et al. [64], who found effects on self-esteem among girls following a school-based PA intervention. Especially interesting are the positive effects among girls on the HRQoL domains physical well-being, psychological well-being and autonomy and parents, in addition to their vitality. Improvement of these HRQoL domains might serve as a protector towards negative body image and body dissatisfaction in an age group with perceived pressure of achieving a certain type of body and appearance [65]. In this study, the negative effect on sedentary time among girls is a result of an improvement in reduced sedentary time amongst girls in the control group. The girls from the intervention group did not increase their sedentary time during the study period nor did they significantly reduce their sedentary time. This program did not demonstrate any effectiveness for reducing pupils’ sedentary time on a full day or during school time in the short timeframe where pupils wore the accelerometers. The lack of results in reducing sedentary time in secondary schools is in accordance with a recently published pilot study from the UK, who found no evidence of reduced sedentary time after implementing PAL [55]. This indicates the importance of examining levels of PA and sedentary time as individual and independent constructs [66].

The findings are strengthened by use of an objective assessment of PA and physical fitness, as well as by validated instruments for assessment of vitality and HRQoL. The implementation by the Telemark County Council and the naturalistic setting increases the external validity of the findings. The non-randomized design is a limitation, and the power and sample size were small. Yet, this makes the statistically significant findings even more robust. Unfortunately, the delivery of the intervention is not systematically documented, and the naturalistic setting provide natural variations both within and between schools. Hence, the naturalistic setting is also a limitation to the internal validity of the results.

The results of this study should be viewed in light of the mentioned limitations. However, implications of the findings include the need for long-term follow-up in order to examine sustainability of the effects. Furthermore, examining the choice of activities and organizational forms during PAL lessons will provide more in-depth knowledge about the PA behavior in PAL. 

## 5. Conclusions

The seven-month Active and Healthy Kids program led to overall increased school-based PA and MVPA and further improved physical fitness, vitality and HRQoL among adolescents. The program did not positively influence total PA or total MVPA levels and did not show efficacy in reducing total sedentary time or sedentary time spent in school. Further, the program seemed to benefit girls and boys in different ways.

## Figures and Tables

**Table 1:** PAL competence development of teachers.

Before Start	Follow-up Training
One-day course on how to teach PAL and inspiration to 5-min physical active breaks.	One-day follow-up course on how to facilitate activities indoor.
Afternoon meeting at the local school planning PAL	Afternoon meeting at the local school to share experiences and plan PAL
(mandatory, however not all teachers participated).	(mandatory, however not all teachers participated).

Note: PAL = physical active learning.

**Table 2:** Demographic data at baseline. Data are presented as means (±SE).

	n	Intervention M	n	Control M	*p*-Values
Demographics					
Age (years)	197	13.2 (0.0)	447	13.2 (0.0)	0.13
Sex (% girls/boys)	197	47/53	447	51/49	0.314
Anthropometry					
Body mass (kg)	197	52.5 (0.7)	447	52 (0.5)	0.503
Height (cm)	197	162.2 (0.6)	447	161.3 (0.3)	0.15
BMI (kg/m^2^)	197	19.8 (0.2)	447	20.0 (0.2)	0.472
PA full day					
Total PA (CPM)	197	492.2 (13.2)	447	466.2 (9.8)	0.121
SED (min/day)	197	543.2 (5.7)	447	535.8 (3.9)	0.266
MVPA (min/day)	197	56.1 (1.7)	447	52.3 (1.2)	0.067
PA school time					
Total PA (CPM)	197	440.7 16.3)	447	403.8 (12.5)	0.099
SED (min/day)	197	175.3 (3.2)	447	177.1 (2.4)	0.674
MVPA (min/day)	197	17.6 (0.8)	447	16.4 (0.6)	0.268
Physical fitness					
CRF (m)	197	1005.1 (8.1)	447	990.5 (6.6)	0.173
Strength (cm)	197	163.4 (1.8)	447	161.5 (1.3)	0.423
HRQoL					
Physical well-being	197	46.3 (0.6)	447	46.3 (0.5)	0.981
Psychological well-being	197	51.1 (0.6)	447	50.3 (0.5)	0.369
Autonomy and parents	197	53 (0.7)	447	53.3 (0.5)	0.707
Peers and social support	197	51.4 (0.7)	447	51.3 (0.5)	0.840
School environment	197	53.4 (0.7)	447	51.3 (0.5)	0.018 *
Vitality	197	4.7 (0.1)	447	4.8 (0.1)	0.635

Note: * significant baseline difference between the intervention and control group. Abbreviations: SE = standard error, BMI = body mass index, PA = physical activity, cpm = counts per minute, SED = sedentary time; MVPA = moderate-to-vigorous intensity physical activity, CRF = cardiorespiratory fitness, and HRQoL = health-related quality of life.

**Table 3:** Mean (SE) baseline, follow-up and group (intervention–control) differences (SE) with *p*-values indicating significant changes.

	Intervention			Control			Group diff. (SE)	*p*-Value
	Baseline (SE)	Follow-up (SE)	*p*-Value	Baseline (SE)	Follow-up (SE)	*p*-Value		
n	197			447				
BMI (kg/m^2^	19.8 (0.2)	20.2 (0.2)	0.000 **	20.0 (0.2)	20.4 (0.2)	0.007 *	−0.01 (0.1)	0.934
PA full day								
n	197			447				
Total PA (cpm)	492 (13.1)	505 (20.1)	0.512	466 (9.8)	470 (15.6)	0.762	6,6 (9.1)	0.467
SED (min/day)	543 (5.7)	557 (6)	0.057	536 (3.8)	553 (0.9)	0.012 *	−2.68 (3.7)	0.466
MVPA (min/day)	56 (1.7)	55.9 (1.9)	0.916	52 (1.1)	51 (1.4)	0.485	0.76 (1)	0.446
PA school time								
n	197			447				
Total PA (cpm)	492 (13.1)	505 (20.1)	0.512	466 (9.8)	471 (15.6)	0.762	54.7 (9.7)	0.000 **
SED (min/day)	182 (2.6)	175 (3.2)	0.098	190 (2.2)	177 (2.4)	0.000 **	6.5 (2)	0.001 **
MVPA (min/day)	17 (0.8)	17 (10.8)	0.585	16 (0.6)	13 (0.5)	0.000 **	2.8 (0.4)	0.000 **
n	197			447				
CRF (m)	1005 (8.1)	1021 (7.3)	0.076	990 (6.6)	999 (5.3)	0.164	8.7 (4.1)	0.035 *
Strength (cm)	163 (1.8)	171 (1.8)	0.000 **	161 (1.3)	167 (1.4)	0.000 **	2 (0.5)	0.000 **
HRQL								
n	197			447				
Physical	46 (0.6)	47 (0.7)	0.366	46.3 (0.5)	46.1 (0.5)	0.648	0.9 (0.4)	0.012 *
Psychological	51 (0.6)	51 (0.8)	0.845	50.3 (0.5)	49.0 (0.6)	0.023 *	1.19 (0.4)	0.001 **
Autonomy	53 (0.7)	55 (0.8)	0.001 **	53.3 (0.5)	54.7 (0.6)	0.026 *	1.2 (0.4)	0.004 *
Peers and social	51 (0.7)	50 (0.9)	0.085	51.2 (0.5)	50 (0.5)	0.031 *	−0.4 (0.4)	0.909
School	53 (0.7)	53 (0.8)	0.621	51.3 (0.5)	49.9 (0.6)	0.016 *	2 (0.4)	0.017 *
n	197			447				
Vitality	4.7 (0.1)	4.8 (0.1)	0.412	4.8 (0.0)	4.5 (0.1)	0.000 **	0.3 (0.0)	0.000 **

Note: * statistically significant difference, *p* < 0.05; ** statistically significant, *p* < 0.001. Abbreviations: SE = Standard Error, BMI = body mass index, PA = physical activity, cpm = counts per minute, SED = sedentary time; MVPA = moderate-to-vigorous intensity physical activity, CRF = cardiorespiratory fitness, and HRQoL = health-related quality of life in the five domains, namely physical well-being, psychological well-being, autonomy and parent relationship, peers and social support and school environment.

**Table 4:** Effects for the whole sample.

	Group					Time					Group x Time		
	df	F	*p*	Mean diff (SE)	95% CI	df	F	*p*	Mean diff (SE)	95% CI	df	F	*p*
PA full day													
Total PA (cpm)	538.32	1.44	0.23	0.06 (0.06)	−0.06–0.19	425.27	1.26	0.26	−0.02 (0.05)	−0.11–0.07	424.27	0.01	0.91
SED (min/day)	508.71	2.3	0.13	0.08 (0.63)	−0.42–0.21	407.00	6.67	0.01 *	−0.06 (0.05)	−0.15–0.03	407.00	0.00	0.97
MVPA (min/day)	510.53	3.09	0.08	0.08 (0.06)	−0.05–0.21	392.52	0.31	0.58	0.03 (0.05)	−0.07–0.12	392.52	0.06	0.80
PA school time													
Total PA (cpm)	554.87	25.23	0.00 **	0.23 (0.06)	0.11–0.34	520.83	3.11	0.08	0.10 (0.04)	0.01–0.19	520.83	7.99	0.005 *
SED (min/day)	5552.18	5.91	0.02 *	−0.12 (0.05)	−0.22–(−)0.01	544.88	11.74	0.00 **	0.08 (0.04)	−0.01–0.16	544.88	1.12	0.29
MVPA (min/day)	536.04	17.17	0.00 **	0.19 (0.06)	0.08–0.31	483.26	9.26	0.00 *	0.12 (0.04)	0.04–0.21	483.26	9.04	0.003 **
CRF (m)	582.85	7.57	0.00 **	0.13 (0.07)	−0.00–0.27	458.72	5.61	0.02 *	−0.03 (0.04)	−0.11–0.06	458.72	5.31	0.02 *
Strength (cm)	609.17	1.12	0.29	0.04 (0.07)	−0.1–0.18	469.02	113.55	0.00 **	−0.14 (0.04)	−0.23–(−)0.05	469.02	3.87	0.050 *
HRQoL													
Physical	621.19	0.39	0.53	0.03 (0.06)	−0.1–0.15	566.23	0.58	0.45	−0.02 (0.04)	−0.10–0.07	566.23	1.35	0.25
Psychological	621.84	4.15	0.04 *	0.01 (0.06)	−0.03–0.22	566.62	2.28	0.13	0.05 (0.04)	−0.03–0.14	566.62	1	0.17
Autonomy	619.73	0.14	0.71	0.02 (0.06)	−0.1–0.13	575.67	13.72	0.00 **	−0.09 (0.04)	−0.18–(−)0.00	575.67	1.77	0.26
Peers	606.21	0.05	0.82	0.01 (0.06)	−0.11–0.13	560.06	4.09	0.04 *	0.05 (0.04)	−0.04–0.14	560.06	0.00	0.99
School	625.08	13	0.00 **	0.17 (0.06)	0.05–0.28	578.53	2.42	0.12	0.08 (0.04)	−0.01–0.16	578.53	1.38	0.24
Vitality	608.53	1.61	0.22	0.05 (0.06)	−0.07–0.18	550.09	1.64	0.20	0.03 (0.04)	−0.05–0.12	550.09	7.20	0.008 **

Note: Group = intervention and control, Time = baseline measurements and follow-up. Pupils and schools were included as random effects to account for clustering. * statistically significant difference, *p* < 0.05; ** statistically significant, *p* < 0.001. Abbreviations: CI = confidence interval, SE = standard error, PA = physical activity, cpm = counts per minute, SED = sedentary time; MVPA = moderate-to-vigorous intensity physical activity, CRF = cardiorespiratory fitness, HRQoL = health-related quality of life in the five domains, namely physical well-being, psychological well-being, autonomy and parent relationship, peers and social support and school environment.

**Table 5:** Effects for boys.

	Group					Time					Group x Time		
Boys	df	F	*p*	Mean diff (SE)	95% CI	df	F	*p*	Mean diff (SE)	95% CI	df	F	*p*
PA full day													
Total PA (cpm)	252.01	0.02	0.88	0.08 (0.09)	−0.10–0.26	185.93	2.26	0.14	0.13 (0.07)	0.00–0.27	185.93	0.09	0.758
SED (min/day)	235.15	3.90	0.05 *	0.09 (0.09)	−0.09–0.26	204.09	9.41	0.00 *	−0.18 (0.07)	−0.32- (-)0.04	204.09	0.04	0.842
MVPA (min/day)	246.53	0.32	0.57	0.14 (0.10)	−0.05–0.33	197.17	2.99	0.09	0.19 (0.07)	0.04–0.33	197.17	0.80	0.373
PA school day													
Total PA (cpm)	288.57	10.96	0.00 **	0.43 (0.09)	0.25–0.61	267.23	2.08	0.15	0.31 (0.07)	0.17–0.45	267.23	9.71	0.002 *
SED (min/day)	281.09	1.69	0.19	−0.24 (0.08)	−0.39–(−)0.09	271.58	4.37	0.04 *	−0.08 (0.07)	−0.21–0.05	271.58	0.44	0.508
MVPA (min/day)	265.66	8.90	0.00 *	0.34 (0.08)	0.18–0.51	235.39	5.29	0.02 *	0.28 (0.07)	0.15–0.41	235.39	13.05	0.000 **
CFR (m)	293.27	9.21	0.00 *	0.45 (0.10)	0.26–0.64	236.81	7.22	0.01 *	0.20 (0.06)	0.07–0.32	236.81	1.46	0.229
Strength (cm)	305.96	6.08	0.01 *	0.38 (0.10)	0.179	243.62	74.62	0.00 **	0.06 (0.06)	−0.06–0.19	243.62	1.85	0.175
HRQoL													
Physical	315.26	0.21	0.64	0.04 (0.09)	−0.13–0.21	290.05	2.73	0.20	0.01 (0.06)	−0.11–0.13	290.05	0.11	0.744
Psychological	308.20	0.04	0.84	0.08 (0.08)	−0.09–0.24	282.88	0.01	0.93	0.06 (0.06)	−0.05–0.18	282.88	0.03	0.868
Autonomy	313.08	0.31	0.58	0.12 (0.08)	−0.15–0.07	293.77	12.70	0.00 **	−0.15 (0.06)	−0.27–(−)0.02	293.77	0.07	0.785
Peers	304.80	1.31	0.25	−0.17 (0.09)	−0.34–(−)0.00	283.45	1.29	0.26	−0.07 (0.06)	−0.20–0.05	283.45	0.05	0.830
School	313.29	6.76	0.01 *	0.14 (0.08)	−0.02–0.30	293.12	0.18	0.68	0.02 (0.06)	−0.01–0.1	293.12	0.51	0.474
Vitality	309.28	0.11	0.74	0.08 (0.08)	−0.08–0.25	281.71	0.45	0.51	0.05 (0.06)	−0.07–0.16	281.71	0.58	0.447

Note: Group = intervention and control, Time = baseline measurements and follow-up. Pupils and schools were included as random effects to account for clustering. * statistically significant difference, *p* < 0.05; ** statistically significant, *p* < 0.001. Abbreviations: CI = confidence interval, SE = standard error, PA = physical activity, cpm = counts per minute, SED = sedentary time; MVPA = moderate-to-vigorous intensity physical activity, CRF = cardiorespiratory fitness, HRQoL = health-related quality of life in the five domains, namely physical well-being, psychological well-being, autonomy and parent relationship, peers and social support and school environment.

**Table 6:** Effects for girls.

	Group					Time					Group x Time		
Girls	df	F	*p*	Mean diff (SE)	95% CI	df	F	*p*	Mean diff (SE)	95% CI	df	F	*p*
PA full day													
Total PA (cpm)	286.85	1.95	0.16	0.05 (0.09)	−0.13–0.23	237.49	8.12	0.01 *	−0.25 (0.07)	−2.28–(−)0.02	237.49	0.01	0.916
SED (min/day)	272.79	0.06	0.80	0.07 (0.09)	−0.10–0.25	207.65	0.44	0.51	0.036 (0.06)	−0.09–0.16	207.65	0.00	0.973
MVPA (min/day)	266.5	3.51	0.06	0.03 (0.08)	−0.14–0.19	200.60	0.94	0.33	−0.12 (0.06)	−0.22–0.01	200.60	0.22	0.637
PA school time													
Total PA (cpm)	275.84	13.4	0.00 **	−0.00 (0.07)	−0.14–0.13	256.61	1.56	0.21	−0.11 (0.05)	−0.21–(−)0.01	256.61	0.25	0.697
SED (min/day)	518	3.41	0.07	0.03 (0.07)	−0.12–0.17	518	7.23	0.01 *	0.23 (0.06)	0.11–0.35	518	5.34	0.021 *
MVPA (min/day)	274.35	6.89	0.01 *	0.014* (0.08)	−0.14–0.17	253.28	4.93	0.03 *	−0.23 (0.06)	−0.12–0.09	253.284	0.18	0.672
CFR (m)	287	0.34	0.56	−0.22 (0.09)	−0.39–(−)0.43	221.88	0.33	0.57	−0.26 (0.06)	−0.38–(−)0.15	221.88	5.23	0.023 *
Strength (cm)	299.50	2.32	0.13	−0.34 (0.09)	−0.52–(−)0.15	222.95	39.75	0.00 **	−0.36 (0.06)	−0.47–(−)0.25	222.95	2.15	0.144
HRQoL													
Physical	304.15	1.68	0.20	0.01 (0.09)	−0.17–0.18	273.98	0.38	0.54	−0.04 (0.06)	−0.16–0.08	273.98	4.13	0.043 *
Psychological	310.94	6.80	0.01 *	0.11 (0.09)	−0.06–0.29	280.61	5.00	0.03 *	0.05 (0.06)	−0.08–0.17	280.61	4.5	0.035 *
Autonomy	303.45	0.00	0.98	0.02 (0.09)	−0.15–0.19	277.47	2.61	0.11	−0.04 (0.06)	−0.15–0.08	277.47	3.95	0.048 *
Peers	297.63	3.12	0.08	0.21 (0.08)	0.04–0.38	273.80	0.06	0.81	0.18 (0.06)	0.06–0.3	273.80	0.06	0.807
School	308.58	6.46	0.01 *	0.20 (0.08)	0.03–0.37	281.67	3.61	0.06	0.13 (0.06)	0.01–.25	281.67	0.89	0.348
Vitality	297.27	1.6	0.20	0.01 (0.09)	−0.17–0.20	265.46	5.77	0.02 *	0.02 (0.06)	−0.11–0.15	265.46	8.75	0.003 *

Note: Group = intervention and control, Time = baseline measurements and follow-up. Pupils and schools were included as random effects to account for clustering. * statistically significant difference, *p* < 0.05; ** statistically significant, *p* < 0.001. Abbreviations: CI = confidence interval, SE = standard error, PA = physical activity, cpm = counts per minute, SED = sedentary time; MVPA = moderate-to-vigorous intensity physical activity, CRF = cardiorespiratory fitness, HRQoL = health-related quality of life in the five domains, namely physical well-being, psychological well-being, autonomy and parent relationship, peers and social support and school environment.
